# A Novel Neurotoxin from Venom of the Spider, *Brachypelma albopilosum*

**DOI:** 10.1371/journal.pone.0110221

**Published:** 2014-10-20

**Authors:** Yunhua Zhong, Bo Song, Guoxiang Mo, Mingwei Yuan, Hongli Li, Ping Wang, Minglong Yuan, Qiumin Lu

**Affiliations:** 1 Faculty of Environmental Science and Engineering, Kunming University of Science and Technology, Kunming, Yunnan, China; 2 Cadres Treatment Section & Thoracic Surgery Department, the First People's Hospital of Yunnan Province, Kunming, Yunnan, China; 3 School of Basic Medical Sciences, Yunnan University of Traditional Chinese Medicine, Kunming, Yunnan, China; 4 College of Humanities & Social Sciences, Nanjing Agricultural University, Nanjing, Jiangsu, China; 5 Engineering Research Center of Biopolymer Functional Materials of Yunnan, Yunnan University of Nationalities, Kunming, Yunnan, China; 6 Key Laboratory of Animal Models and Human Disease Mechanisms of Chinese Academy of Sciences & Yunnan Province, Kunming Institute of Zoology, Kunming, Yunnan, China; University of Würzburg, Germany

## Abstract

Spiders have evolved highly selective toxins for insects. There are many insecticidal neurotoxins in spider venoms. Although a large amount of work has been done to focus on neurotoxicity of spider components, little information, which is related with effects of spider toxins on tumor cell proliferation and cytotoxicity, is available for *Brachypelma albopilosum* venom. In this work, a novel spider neurotoxin (brachyin) was identified and characterized from venoms of the spider, *Brachypelma albopilosum*. Brachyin is composed of 41 amino acid residues with the sequence of CLGENVPCDKDRPNCCSRYECLEPTGYGWWYASYYCYKKRS. There are six cysteines in this sequence, which form three disulfided bridges. The serine residue at the C-terminus is amidated. Brachyin showed strong lethal effects on American cockroaches (*Periplaneta americana*) and *Tenebrio molitor* (common mealbeetle). This neurotoxin also showed significant analgesic effects in mice models including abdominal writhing induced by acetic acid and formalin-induced paw licking tests. It was interesting that brachyin exerted marked inhibition on tumor cell proliferation.

## Introduction

As excellent predators, spiders kill or paralyze their prey by using their venoms, which are secreted from their venom glands. Several families of bioactive substances have been found from spider venoms including low-molecular-weight compounds, peptides, and proteins. These venom components possess functions including induction of paralysis, activation or inhibition of ion channels and receptors, and microorganism-killing [1]. There is extreme chemical diversity in spider venoms, which are a rich resource from which to prospect bioactive compounds. High levels of chemical diversity make spider venoms attractive subjects for chemical prospecting. More than 500 bioactive peptides from venoms of about 60 spider species with molecular weight of less than 10 kDa are characterized and divided into 20 families [1]. Most of these spider peptides contain six or eight cysteine residues to form three to four disulfide bridges but they have different disulfide bond motifs. Most of these disulfide-containing peptides exhibit neurotoxic properties.

*B. albopilosum* is a species of tarantula known commonly as the Honduran curlyhair or simply Curlyhair tarantula. It is distributed in Central America, from Honduras to Costa Rica. They are terrestrial, opportunistic burrowing spiders [2]. *B. albopilosum* is frequently kept and bred as a pet in captivity. Although *B. albopilosum* is one of the commonest spiders in Central America, little work has been done to study compounds in its venoms. A novel neurotoxin is identified from the venom of *B. albopilosum* in this study.

## Materials and Methods

### Animals

Both male and female Kunming mice with weight range from 18 to 22 g were used in the study. Mice were kept under standard acclimatization conditions of 12 h light/dark cycle at 25°C and were provided standard rodent feed procedure. All animal experiments were started after getting approval from the Animal Ethical Committee of the First People’s Hospital of Yunnan province (2013-12) and all guidelines laid down by the Institutional Animal Care and Use Committee (IACUC) were followed during the entire course of the experiment.

### Spider venom collection

Adult female spiders of *B. albopilosum* were purchased from the pet market of Kunming city in Yunnan province of China. The spider venoms were collected according to previously published methods [3]–[6]. Briefly, the venom was obtained by electrical stimulation of female spiders. Then the collected venoms were kept at −20°C.

### Purification of neurotoxin peptide

The spider venom sample (0.2 g) was dissolved in 10 ml of 0.1 M phosphate buffer solution (PBS), pH 6.0 containing 5 mM EDTA (total absorbance at 280 nm was 370 AU). The venom sample was applied to a Sephadex G-50 (Superfine, Amersham Biosciences, 2.6 cm×90 cm, Hong Kong, China) gel filtration column which was equilibrated with 0.1 M PBS (pH 6.0). The elution was performed with the same PBS buffer at 4°C. The absorbance of the elution was monitored at both 215 and 280 nm. Every elution fraction (3.0 ml) was collected in ten minutes and a total elution volume of 540 ml was used for the Sephadex column in 30 hr. The protein peak containing insecticidal activity was pooled, lyophilized, and resuspended in 5 ml 0.1 M PBS (pH 6.0), and purified further with a C8 RP-HPLC (Tigerkin C8, 30 cm×0.21 cm, Dalian Sipore Co., Ltd, Dalian, China) column on a Waters™ 1525 binary pump system (Waters China Ltd., Hong Kong). The elution was performed with determined gradient (0–80% in 70 min) of acetonitrile in 0.1% TFA in H_2_O at a flow rate of 0.7 ml/min.

### Primary structure analysis

The purified peptide was subjected to amino acid sequencing by Edman degradation on an Applied Biosystems pulsed liquid-phase sequencer, model 491. The Cys sulfhydryl group was alkylated by reaction with 4-vinyl pyridine according to the method described by Friedman et al. [7]. The actual molecular mass of the peptide was measured by a Matrix-Assisted Laser Desorption Ionization Time-Of-Flight mass spectrometer (MALDI-TOF-MS, AXIMA CFR (Kratos Analytical)) in positive ion and linear mode. α-Cyano-4-hydroxycinnamic acid (CHCA) was used as matrix. The specific operating parameters were as follows: the ion acceleration voltage was 20 kV; the accumulating time of single scanning was 50 s. The polypeptide mass standard (Kratos Analytical) was used as the external standard.

### cDNA library and cloning

Total RNA was extracted from venom glands of *B. albopilosum* using TRIzol (Life Technologies Ltd.). The total RNA was used to prepare cDNA by using a SMART PCR cDNA synthesis kit purchased from Clontech (Palo Alto, CA). The first strand was synthesized by using the 3′ SMART CDS Primer II A (5′ AAGCAGTGGTATCAACGCAGAGTACT(30)N-1N 3′, where N = A, C, G, or T and N-1 = A, G, or C) and SMART II A oligonucleotide, (5′ AAGCAGTGGTATCAACGCAGAGTACGCGGG 3′). 5′ PCR primer II A (5′ AAGCAGTGGTATCAACGCAGAGT 3′) provided by the kit was used to synthesize the second strand using Advantage polymerase (Clontech, Palo Alto, CA). The synthesized cDNA was used for cDNA library construction. A directional cDNA library was constructed with a plasmid cloning kit (SuperScriptTM Plasmid System, GIBCO/BRL) following the manufacturer’s instructions. A cDNA library was produced with 2.6×10^5^ independent colonies.

A PCR-based method for high stringency screening of DNA libraries was used to screen and isolate cDNA clones. Sense-direction primer (5′ TG(T/C)CT(A/T/G/C)AG(A/T/G/C)GA(A/G)AA(T/C)GT(A/T/G/C)CC(A/T/G/C)TG(T/C)GA3′) was designed according to the amino acid sequence of brachyin, which was determined by Edman degradation. The primer was used in conjunction with an antisense SMART II A primer II in PCR reaction to screen for transcripts encoding brachyin. PCR was performed by using Advantage polymerase (Clontech) according to the condition: 2 min at 94°C, followed by 30 cycles of 10 s at 92°C, 30 s at 55°C, 40 s at 72°C. PCR products were cloned into pGEM-T Easy vector (Promega, Madison, WI). DNA sequencing was performed on an ABI PRISM 377 DNA sequencer (Applied Biosystems).

### Insecticidal test

Purified neurotoxins were dissolved in insect saline, pH 7.4 (mM concentration in deionized water: NaCl 140, KCl 5, NaHCO_3_ 4, MgCl_2_ 1, CaCl_2_ 0.75, HEPES 5) and injected into American cockroaches (*Periplaneta americana*; mass 700–900 mg) and mealbeetles (*Tenebrio molitor* larvae; mass 190–210 mg) (n = 10). The median lethal dose (LD_50_) was determined according to the method described by Tedford et al. [8].

### Abdominal writhing induced by acetic acid

Five groups of mice were selected for the present study. According to the method described by Santos et al [9], groups of mice (n = 10) were injected via the peritoneal route (i.p.) with 100 µl saline containing brachyin (0.1, 0.2 or 0.4 mg/kg), or morphine (5 mg/kg, positive control for analgesic activity) 30 min prior to injection of acetic acid solution (0.8% v/v; 10 ml/kg, i.p.) which induces abdominal contraction and hind limb stretching. The control group received the same volume of 0.9% saline. Experimental animals were placed into open polyvinyl cages (20×40×15 cm) immediately after acid challenge, and abdominal constrictions were counted cumulatively over a period of 30 min.

### Formalin-induced paw licking

The mice were divided into five groups (for each group, n = 10). Pain was induced in mice by intraplantar injection of formalin [10]. Pain attenuation was evaluated after the application of brachyin (0.1, 0.2, or 0.4 mg/kg), or morphine (5 mg/kg, positive control for analgesic activity) dissolved in 100 µl saline by i.p injection in mice. Control mice received the same volume of 0.9% saline. After 30 min pre-treatment by testing sample, animals were injected with formalin (20 µl, 2.5% v/v) under the plantar surface of right hind paw. Mice were then kept individually in open polyvinyl cages (20×40×15 cm). The time spent on licking the injected paw was recorded by a digital video camera during Phase I (0–5 min post-injection) and Phase II (15–30 min post-injection).

### Cell proliferation inhibitory activity

The following cell lines were used to assess the cell proliferation inhibitory activity of brachyin: Human HaCaT keratinocyte cell, human T cell lines Molt-4 and C8166 (Chinese Type Culture Collection, Kunming Institute of Zoology, Chinese Academy of Sciences); the bladder cancer cell lines BIU-87 and T24, lung cancer cell lines A549 and Calu-6 (West China Medical University). All cell lines were maintained in RPMI-1640 (GIBCO) medium supplemented with 10% (v/v) heat-inactivated fetal calf serum (FCS), 2 mM glutamine (Sigma), 10 mM Hepes (Sigma), 50 mM 2-mercaptoethanol (Bio-Rad), 100 units/ml penicillin and 100 µg/ml streptomycin. 100 µl of Molt-4 or C8166 cells (3×10^5^/ml) were seeded into a microtiter plate. For BIU-87, T24, A549 and Calu-6 cell lines, 100 µl of trypsinized cells (2×10^5^/ml) were seeded into a microtiter plate and allowed a preincubation period of overnight at 37°C for attachment. Various concentrations of brachyin were added and incubated for 48 h. Cytotoxicity was measured by the MTT method. Briefly, after adhering to the plate, cells were incubated with vehicle (DMEM) or brachyin at different concentrations for 24 h. Then 20 µl of 3-(4,5-dimethylthiazol-2-yl)-2,5-diphenyl-2H-tetrazolium bromide (MTT) solution (R&D Systems Inc. Minneapolis, MN, USA) was added to each well for a further 4 h incubation at 37°C. After the cells were washed 3 times with PBS (pH 7.4), the insoluble formazan product was dissolved by incubation with 150 µl DMSO. The absorbance of each well was measured on an enzyme-linked immunosorbent assay (ELISA) microplate reader at 490 nm [11]. The optical density reflects the level of cell metabolic activity. Each experiment was performed in quintuplicate. IC_50_ was defined as the concentration of the sample at which the absorbance at 490 nm was reduced by 50%.

### Statistical Analysis

All the data were presented as means ± SEM (standard error of mean) and analyzed by one-way analysis of variance followed by unpaired *t* test. A value of p<0.05 was taken as the level of significance.

## Results

### Purification of spider neurotoxin

**Figure 1:**
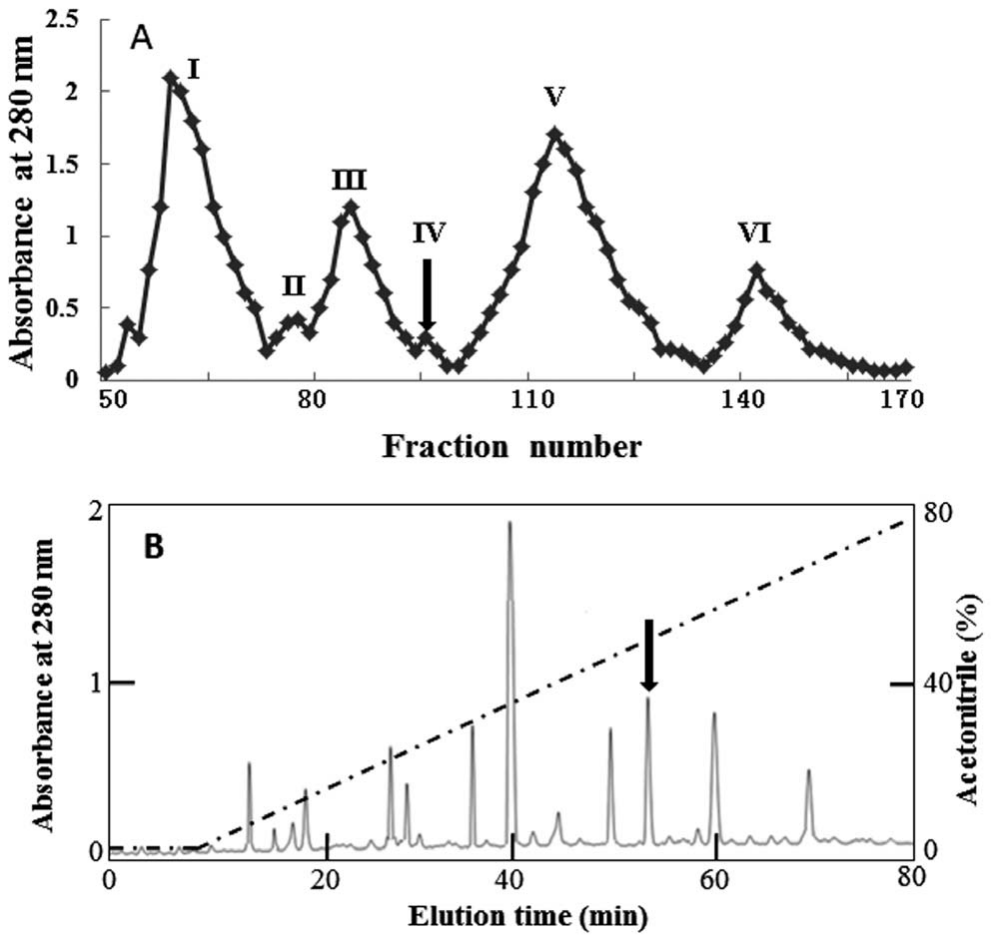
Purification of brachyin from spider venom of *B.* *albopilosum*. A: Sephadex G-50 gel filtration of spider venom of *B. albopilosum*. The spider venom of *B. albopilosum* was applied to a Sephadex G-50 gel filtration column. The elution was performed by 0.1 M PBS at a flow rate of 0.3 ml/min, collecting fractions of 3.0 ml. The fraction containing insecticidal activity is marked by an arrow. B: The interesting fraction from the Sephadex G-50 gel filtration was further purified by a C8 RP-HPLC column. The elution was performed at a flow rate of 0.7 ml/min with the indicated gradient of acetonitrile in 0.1% (v/v) trifluoroacetic acid (TFA) in water. Purified brachyin is indicated by an arrow.

The spider venom was fractionated into six protein peaks by Sephadex G-50 as illustrated in Fig. 1A. Among these eluted fractions, Peak IV (fractions 92–97) was found to exert insecticidal activities (arrowed). Peak IV was collected and purified further by RP-HPLC. More than ten peaks were eluted from the RP-HPLC as illustrated in Fig. 1B. The purified peptide eluted at 53.52 min (marked by an arrow) was named brachyin. Three separate procedures of purification of brachyin were performed and the profiles of the Sephadex G 50 and RP-HPLC were reproducible.

### Structural characterization

Purified brachyin was subjected to amino acid sequence analysis by automated Edman degradation. The amino acid sequence of brachyin was determined as CLGENVPCDKDRPNCCSRYECLEPTGYGWWYASYYCYKKRS with an observed mass of 4901.7 determined by mass spectrometry. Treating with carboxypeptidase Y did not lead to the release of free amino acids under conditions that free amino acids were released from a peptide with free C-terminal -COOH group. The result indicated that the C-terminal end of the peptide was amidated. There actually is the amidation signal G in the C-terminal, which is cleaved at Cα atom by amidating enzymes to yield the C-terminal amidation. The observed mass (4901.7) is matched well with the theoretical molecular weight (4901.5 Da) of brachyin containing three intra-molecular disulfided bridges and an amidated C-terminus.

### cDNA cloning

**Figure 2:**
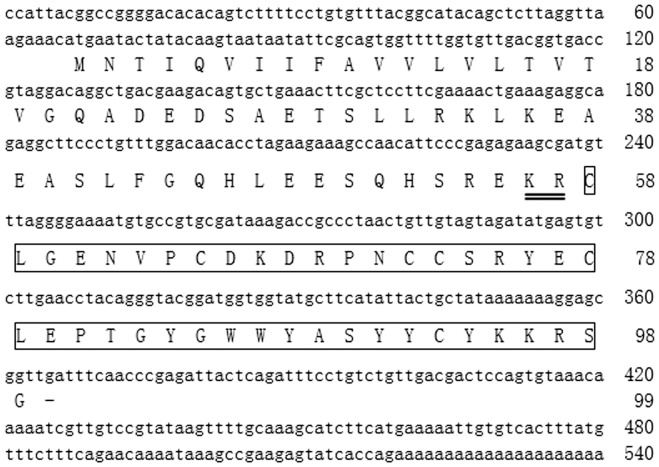
cDNA sequence encoding brachyin and deduced amino acid sequence. Mature peptides are boxed. The bar (−) indicates stop codon. The di-basic site for proprotein convertase processing is double lined.

**Figure 3:**
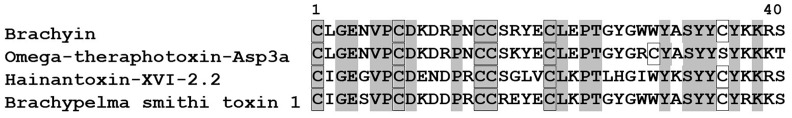
Sequence alignment of precursors encoding spider venom neurotoxin peptides. Identical amino acid residue are in shade. Cysteines that may form disulfide bonds are boxed.

A cDNA clone encoding the precursor of brachyin was screened and sequenced from the venom gland cDNA library of *B. albopilosum*. The cDNA sequence and deduced amino acid sequence is showed in Fig. 2. It encoded a precursor composed of 99 amino acid residues. The structural organization of the precursors is comprised of a signal peptide sequence, an N-terminal spacer peptide region containing multiple aspartic and glutamic acid residues, and a mature brachyin at the C-terminus. The amino acid sequence deduced from the cDNA sequence encoding brachyin is identical to the sequence determined by Edman degradation. By BLAST search, it was found to share significant sequence homology with other spider neurotoxin peptides including Brachypelma smithi toxin 1 from the spider *Brachypelma smithi* (identity 78.0%) [12], HNTX-XVI-2.2 from the spider *Ornithoctonus hainana* (identity 70.7%) [4], and Omega-theraphotoxin-Asp3a from the spider *Aphonopelma sp.* (identity 85.4%) (UniProtKB/Swiss-Prot: P0CI04.1) (Fig. 3). There is a di-basic site for proproteinconvertases processing between the spacer peptide region and mature brachyin as other precursors of spider neurotoxin peptides (Fig. 2).

### Insecticidal activity

The lethal activity of brachyin against cockroaches and mealbeetles was tested. It had potent insecticidal activity. Ten min to 2 h following injection of the neurotoxin, these insects showed signs of neurotoxicity including twitching, paralysis, and body contraction. As shown in Table 1, the neurotoxin dose-dependently exerted its lethal toxicity on each of the tested insects. Brachyin killed all of the tested cockroaches and mealbeetles at a dosage of 12.0 µg/kg and 15.0 µg/kg, respectively. The LD_50_ of brachyin is 5 µg/kg in adult cockroaches and 7.6 µg/kg in mealbeetles calculated by Probit regression in the SPSS software.

**Table 1:** The insecticidal activity of brachyin.

Brachyin (µg/kg)	Total cockroach^a^	Dead cockroach^b^	Total mealbeetle^a^	Dead mealbeetle^b^
0.0	10	0±0.0	10	0±0.0
1.0	10	0±0.0	10	0±0.0
3.0	10	2.3±0.6	10	1.3±0.5
6.0	10	6.6±0.9	10	3.3±0.5
9.0	10	9.3±0.5	10	6.3±0.8
12.0	10	10.0±0.0	10	8.6±0.9
15.0	10	10.0±0.0	10	10.0±0.0

Brachyin was dissolved in insect saline and injected into American cockroaches (*Periplaneta americana*; mass 700–900 mg) and mealbeetles (*Tenebrio molitor* larvae; mass 190–210 mg). The death of the insects was counted.

a For each dosage of brachyin, a total of 10 insects were tested.

b Data are means±SEM of triplicate determinations (n = 10).

### Analgesic activities

**Figure 4:**
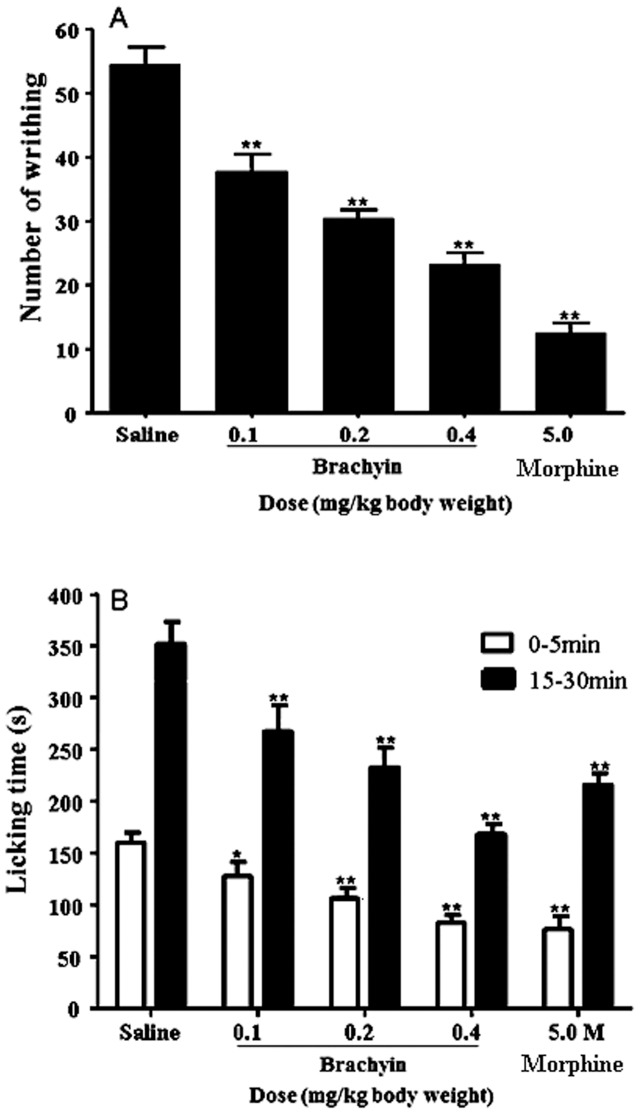
Analgesic activities of brachyin in the mice model of acetic acid-induced abdominal writhing (A) and formalin-induced paw licking (B). M: morphine. Data are means±SEM. Significant difference compared with control group treated by 0.9% saline is indicated by *(*p*<0.05) or **(*p*<0.01) (n = 10).

The analgesic effects of brachyin were tested in two mice pain models in which pain was induced by noxious chemicals or inflammatory response. As illustrated in Fig. 4A, brachyin reduced the abdominal writhing induced in mice by i.p. injection of acetic acid. Compared with the number (54±4) of writhing movements induced by acetic acid in control group, brachyin application at the dosage of 0.1, 0.2 and 0.4 mg/kg decreased the number to 38±4, 31±2 and 24±3, respectively. The number in the positive control of morphine (5 mg/kg) was 13±2.

Intraplantar injection of formalin leads to a biphasic pain response in mice comprising early neurogenic pain (0–5 min) that is followed later by inflammatory pain (15–30 min). Intraperitoneal application of brachyin markedly decreased both neurogenic and inflammatory pain responses (Fig. 4B). Control mice (0.9% saline injection) licked their paws with an average of ∼160 s during the early phase. The average licking time after brachyin application at the dosage of 0.1, 0.2 and 0.4 mg/kg was ∼126, 107 and 80 s, respectively. The time for morphine (5 mg/kg) application was 75 s. During the second phase, control mice licked their paws with an average of ∼355 s. The time for brachyin (0.1, 0.2 and 0.4 mg/kg) application was ∼270, 235 and 170 s, respectively. The time for morphine (5 mg/kg) application was 215 s.

### Cell proliferation inhibition

**Figure 5:**
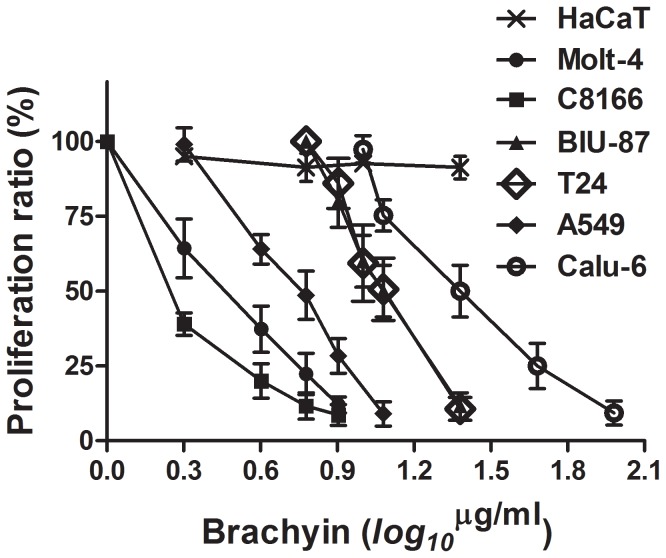
Cell proliferation inhibitory activity of brachyin. Human HaCaT keratinocyte cell, human T cell lines Molt-4 and C8166, bladder cancer cell lines BIU-87 and T24 and lung cancer cell lines A549 and Calu-6 were seeded into a microtiter plate and allowed a preincubation period of overnight at 37°C for attachment. Various concentrations of brachyin were added and incubated for 48 h. Cytotoxicity was measured by the MTT method. Means ± SEM of four experiments. *P<0.05 vs 0 brachyin.

Brachyin possessed significant cell proliferation inhibition activity in all tumor cell lines tested (Fig. 5). MTT assays of the six human tumor cell lines revealed that IC_50_ value against C8166, Molt-4, A549, BIU-87, T24, and Calu-6 cell line was 1.5, 3.0, 6, 12, 12, and 24 µg/ml, respectively. Comparatively, human tumor cells especially those of solid tumor cell lines (Calu-6, BIU-87 and T24) were less sensitive to brachyin. Generally, C8166 cells were more sensitive than other cell lines to this peptide. Brachyin had no effect on human HaCaT keratinocyte cell proliferation even at a very high concentration of 98 µg/ml (data not shown).

## Discussion

The compositions of spider venom components are very complex. They are mixtures of biologically active compounds with different chemical natures. More than a hundred different components can be found in the same venom. These venom components work synergistically and provide efficiency of action of the mixture [1]. Venom composition is highly species-specific and depends on many factors including sex, nutrition, natural habitat, climate, etc. [13]–[21]. Most spider venoms contain multiple disulfide-containing peptide neurotoxins, which are predominant components of venoms. More than 500 peptides with molecular weight <10 kDa [22] have been characterized from more than 60 spider species. Most of these peptides contain disulfide bonds and exhibit neurotoxic functions. They interact with Na^+^, K^+^, and Ca^2+^ channels, and H^+^, mechano-, and thermo- receptors [1].

Venoms from *B. albopilosum* has been found to exert immobilizing and lethal effects on the cockroach and the common mealbeetle [23] but no information related with the identification and characterization of venom components from this species is available. A novel neurotoxin named brachyin is identified and characterized from the venom of *B. albopilosum* (Fig. 1 & 2). It shares substantial sequence homology with some venom-derived neurotoxins from several other species of spider (Fig. 3). As other spider neurotoxins, brachyin shows insecticidal and analgesic activities (Table 1 & Fig. 4).

It is interesting that brachyin markedly inhibited tumor cell proliferation. Gao et al have found that venom from the spider *Macrothele raven* inhibited cell proliferation and showed cytotoxicity in HeLa cells by induction of apoptosis, necrosis of toxicity damage and direct lysis. However, there was no information on the components in the venom that induced these effects in the report [24]. In the present study, phase contrast photomicrographs showed that brachyin did not induce necrosis of toxicity damage and direct lysis in tumor cell lines (figure not shown).

A recent study indicated that lycosin-I, a peptide from the spider *Lycosa singoriensis* displays both a strong ability to inhibit cancer cell growth *in vitro* and could suppress tumor growth *in vivo* through mitochondrial death pathways to sensitize cancer cells for apoptosis, as well as up-regulating p27 to inhibit cell proliferation [25]. PST, a natural molecule isolated from the lily spider *Pancratium littorale*, was shown to have apoptotic effects specifically upon tumor cells, and not upon their homologous non-tumoral lines by inducing permeabilization of mitochondria and activation of caspases [26]. It is possible that brachyin may inhibit cancer cell growth in a similar way to the peptides listed above. Brachyin may be a good candidate in the development of anti-cancer therapies. Further work is necessary to study the mechanisms of the inhibition of cell proliferation.
